# Evaluating coverage bias in next-generation sequencing of *Escherichia coli*

**DOI:** 10.1371/journal.pone.0253440

**Published:** 2021-06-24

**Authors:** Samantha Gunasekera, Sam Abraham, Marc Stegger, Stanley Pang, Penghao Wang, Shafi Sahibzada, Mark O’Dea

**Affiliations:** 1 Antimicrobial Resistance and Infectious Disease Laboratory, Murdoch University, Western Australia, Australia; 2 Statens Serum Institut, Copenhagen, Denmark; 3 Department of Primary Industries and Regional Development, Diagnostic and Laboratory Services, Western Australia, Australia; CSIR- Institute of Himalayan Bioresource Technology, INDIA

## Abstract

Whole-genome sequencing is essential to many facets of infectious disease research. However, technical limitations such as bias in coverage and tagmentation, and difficulties characterising genomic regions with extreme GC content have created significant obstacles in its use. Illumina has claimed that the recently released DNA Prep library preparation kit, formerly known as Nextera Flex, overcomes some of these limitations. This study aimed to assess bias in coverage, tagmentation, GC content, average fragment size distribution, and *de novo* assembly quality using both the Nextera XT and DNA Prep kits from Illumina. When performing whole-genome sequencing on *Escherichia coli* and where coverage bias is the main concern, the DNA Prep kit may provide higher quality results; though *de novo* assembly quality, tagmentation bias and GC content related bias are unlikely to improve. Based on these results, laboratories with existing workflows based on Nextera XT would see minor benefits in transitioning to the DNA Prep kit if they were primarily studying organisms with neutral GC content.

## 1. Introduction

The introduction of whole-genome sequencing to research in the life sciences has propelled a paradigm shift in diagnosing and managing human and animal infectious diseases. Initially restricted to large research laboratories due to prohibitive costs, this technology has become widespread in smaller diagnostic facilities, allowing for the development of pipelines aimed at rapid disease diagnosis and characterisation of infectious agents at an improved scale and resolution [1]. It is now widely used in outbreak investigations of infectious agents in both medical and veterinary settings [2], understanding the mechanisms of transmission host-jump and evolution of pathogenic microorganisms [3, 4], genomic characterisation of mobile genetic elements responsible for transmission of antimicrobial resistance genes, and vaccine development [5, 6].

Among the second generation of sequencing technologies, the Illumina platforms continue to be the most commonly used [3–6]. Sequencing using Illumina chemistry requires purified genomic DNA to be processed using a library preparation kit, which takes genomic DNA as an input and produces amplified, purified, sequencing-ready DNA libraries [7]. Illumina has released a variety of library preparation kits for different applications, with Nextera XT being widely used for small genomes. The Nextera XT library preparation kit uses a modified transposase that has been engineered to fragment and insert adapters onto genomic DNA in a five-minute reaction that Illumina has coined tagmentation [8].

Studies comparing the Nextera and Nextera XT library preparation kits against other market leaders that do not use enzymatic fragmentation have had mixed conclusions on potential biases introduced by the system [8–13]. The most widely reported concerns have been linked to GC content related biases resulting from the PCR steps required for library amplification [10, 14–20]. There has also been evidence of negative impacts on *de novo* assembly quality [11]. To a lesser degree, insertion biases caused by the modified transposase responsible for tagmentation have also been observed [8, 14, 16, 21]. The presence of coverage bias in a sequencing run can dramatically reduce the amount of information available for analysis and can result in the absence of important loci in the assembled genomes, as well as a loss of important single nucleotide variants [11, 14, 19]. Uniform sequencing coverage of adequate depth can be paramount to the successful characterisation of a bacterial genome [9].

To address the issues associated with Nextera XT, Illumina launched the new Nextera Flex library preparation kit in late 2017 (rebranded in 2020 to DNA Prep) [22]. DNA Prep uses a modified bead-linked transposome, claimed to decrease bias in tagmentation and uses an inbuilt fragment size normalisation mechanism. The bead-linked transposomes remove the need for a strict input DNA volume requirement by only tagmenting DNA after the bead is saturated with DNA. This study aimed to determine whether DNA Prep outperformed Nextera XT when used for the sequencing of *Escherichia coli*, in reducing coverage bias, tagmentation bias and GC content related bias, in addition to producing libraries with a more even fragment size distribution and better downstream *de novo* assembly quality.

## 2. Materials and methods

*Escherichia coli* isolates included in this study originated from faecal samples collected from feral pigeons (*Columba livia*) and little penguins (*Eudyptula minor*) as part of a separate study [23]. Samples (n = 16) were recovered from storage at -80°C by culture onto Columbia sheep blood agar (Micromedia), followed by overnight incubation at 37°C. Following recovery from frozen stock, species identification was confirmed using matrix-assisted laser desorption/ionisation time-of-flight (MALDI-TOF) mass spectrometer (Bruker).

Following species confirmation, DNA extraction was performed using the MagMAX-96 DNA Multi-Sample Kit (Thermo Fisher Scientific) with the following amendments to the manufacturer’s instructions: Five isolated single colonies from each sample were picked and suspended in 200 μL Multi-Sample DNA lysis buffer. All plate shaking steps were performed using a Compact Digital Microplate Shaker (Thermo Fisher Scientific) set to 900 RPM, and an additional plate shaking step was performed following colony addition to the lysis buffer. The MagMAX Express-96 Deep Well Magnetic Particle Processor (Life Technologies) was used with a modified version of the 4413021 DW Blood protocol where the RNase A step was omitted. DNA was eluted in 30 μL of each of the supplied elution buffers. DNA purity was assessed using a NanoQuant Plate (TECAN) and Spark Multimode Microplate Reader (TECAN). The DNA concentration was calculated using a Qubit dsDNA HS Assay kit (Invitrogen).

Library preparation was performed on extracted genomic DNA from all samples (n = 16) with both the Nextera XT library prep kit (Illumina) and the DNA Prep library prep kit (Illumina) according to manufacturer’s instructions. The average fragment size, defined as insert length plus adapter length, for each sample was calculated prior to pooling in preparation for whole-genome sequencing with the LabChip GX Touch HT Nucleic Acid Analyzer. This data was then tested for a statistical significance retrospectively using a paired *t*-test (α of 0.05) to establish whether the fragment size distribution was different depending which library preparation kit was used.

Samples were sequenced on an Illumina NextSeq 500 platform using a V2 mid-output 300-cycle flow cell to obtain paired end 150 bp reads. FASTQ files were downloaded from BaseSpace after being de-multiplexed and subject to adapter trimming. This sequencing run produced two sets of FASTQ files per isolate, with one file corresponding to the Nextera XT library preparation kit and the other corresponding to the DNA Prep library preparation kit. Quality control was performed on all FASTQ files using FastQC v0.11.7, which reported quality metrics including per base sequence quality, per tile sequence quality, per sequence quality scores, per base sequence content, per sequence GC content, per base N content, sequence length distribution, sequence duplication levels, overrepresented sequences, and adapter content [24]. After quality control, the FASTQ files were *de novo* assembled under default settings using SPAdes genome assembler v3.12.0 [25].

For quality assessment of the *de novo* assemblies produced by SPAdes, QUAST v5.0.2 provided a set of metrics including number of contigs, size of the largest contig, total length of assembly and N50 [26]. These values were used to determine whether there was any difference in *de novo* assembly quality that could be attributed to which library preparation kit was used. A paired *t*-test was used for statistical analysis of this data (α of 0.05).

The potential for tagmentation bias was also explored using FastQC with the per base sequence content metric, which calculated the average proportion of each of the four bases at each read position within a FASTQ file [24]. The FASTQ files were merged based on which library preparation kit was used, producing one FASTQ file for the Nextera XT samples and one for the DNA Prep samples. Each merged FASTQ file was parsed through FastQC, and the per base sequence content output was compared for each library preparation kit to confirm whether either of the tagmentation enzymes favoured a sequence motif.

To investigate whether there were any gaps in coverage that were unique to either of the library preparation kits, each set of FASTQ files were mapped to the *E*. *coli* K12 MG1655 reference genome (accession number: NC_000913.3) using Bowtie2 v2.3.4.1 under default settings [27, 28]. The resulting output from each isolate was two alignment files corresponding to each of the library preparation kits. Using SAMtools v1.9, each alignment file was then compressed and sorted before a coverage count at each position across the reference genome for each sample was computed [29]. All statistical analyses were performed using RStudio v1.1.456 [30]. The coverage counts were explored graphically using the R package, ggplot2 v3.2.0 to visually determine regions of low coverage and establish whether the extent of low coverage differed depending on which library preparation kit was used [31]. Initially, a frequency histogram was produced using the coverage count data from each alignment file to observe whether there were more positions of low coverage that corresponded to one library preparation kit over the other. To determine whether there were specific regions of low coverage across the reference genome that were unique to one library preparation kit, the coverage data was also plotted on a bar graph with the position in the reference genome on the x-axis and coverage count on the y-axis. The percentage of the reference genome covered at a given read depth was also explored graphically using ggplot2 v3.2.0 [31]. After outlier removal, the mean coverage counts for each alignment file and the total number of reads for each FASTQ file were tested for statistical significance using a paired *t*-test. In a representative subset of samples (n = 3, accession numbers: SAMN14395278, SAMN14395266, SAMN14395270), regions of low coverage (≤ 5) in the BAM files that were only present in either the DNA Prep or Nextera XT prepared counterpart had GC content percentage calculated. Low coverage regions spanning less than 10 bp were removed. The remaining low coverage data was then plotted using ggplot2 v3.2.0 [31].

## 3. Results

### 3.1 Coverage bias

**Fig 1:**
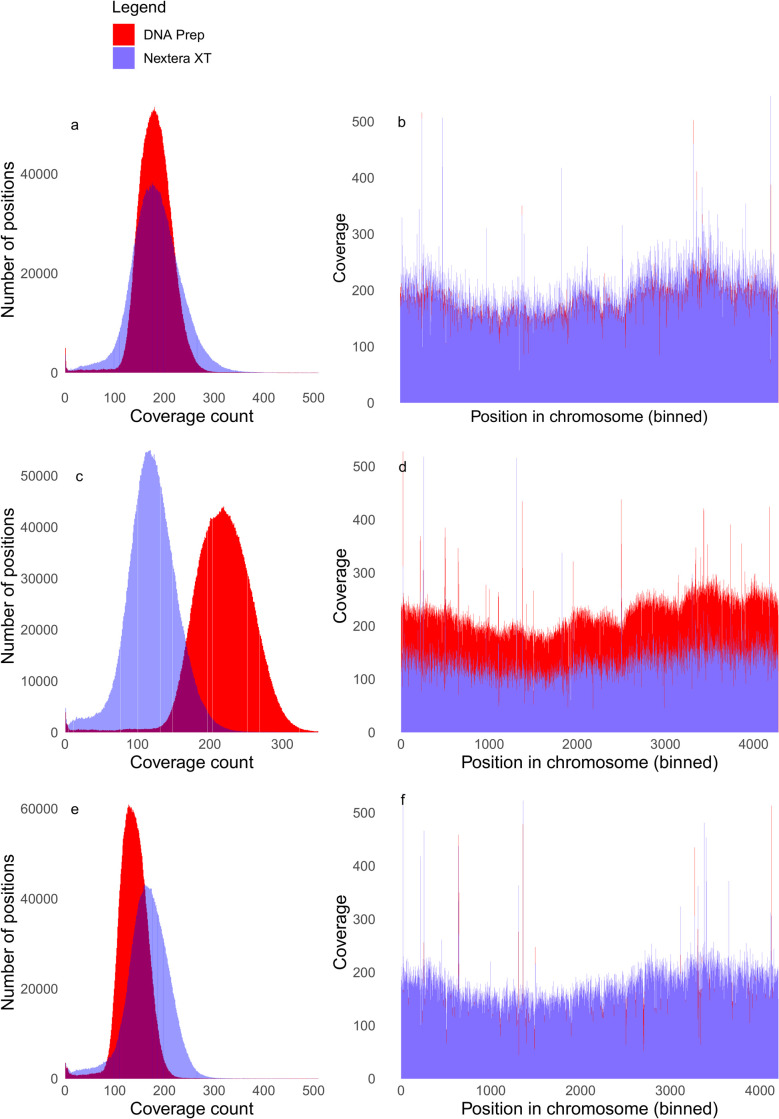
Differences in uniformity of coverage between the Nextera XT and the DNA Prep kits. (A, C, E) Coverage count data from three representative samples displayed as frequency histograms where mean coverage was very similar between the two kits (A), mean coverage was higher in the DNA Prep kit than the Nextera XT kit (C) and mean coverage was higher in the Nextera XT kit than the DNA Prep kit (E). (B, D, F) Coverage count data displayed as bar plots from three representative samples where the DNA Prep dataset showed more uniform coverage than the Nextera XT dataset despite mean coverage being comparable between the two kits (B), which was also the case where mean coverage was higher in the DNA Prep kit than the Nextera XT kit (D) and where mean coverage was higher in the Nextera XT kit than the DNA Prep kit (F).

**Fig 2:**
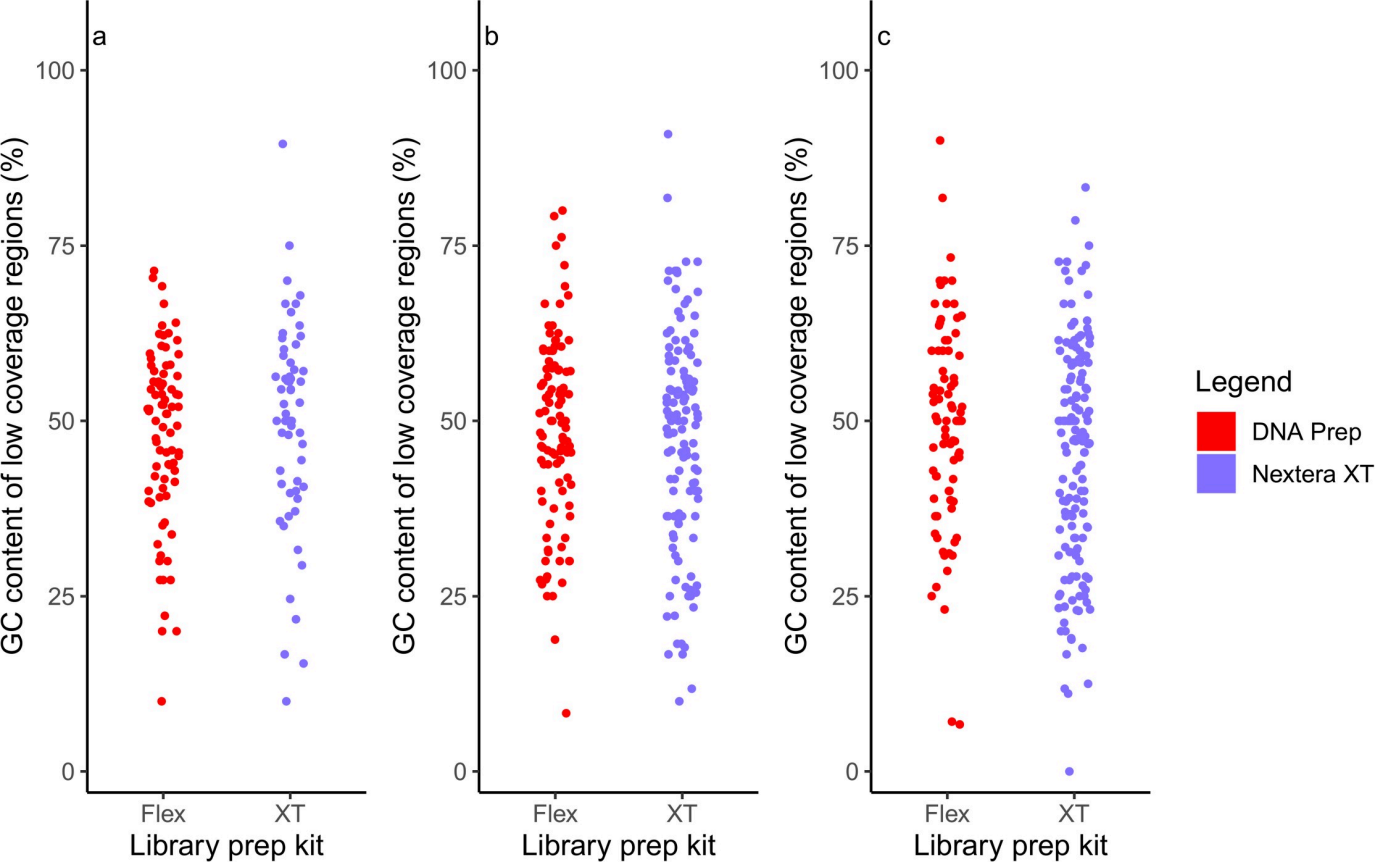
The relationship between GC-content and low coverage regions of the *E*. *coli* chromosome. (A-C) Graph displays the GC-content percentage of low coverage regions (coverage less than 5, each region of length greater than 10 bp) of each representative *E*. *coli* sample.

The DNA Prep library preparation kit yielded whole-genome sequencing data with more uniform coverage than the Nextera XT library preparation kit. In samples where there was no significant difference in mean coverage irrespective of which library preparation kit was used, DNA Prep coverage was more tightly distributed with less variation in contrast to the Nextera XT prepared sample which demonstrated a higher frequency of low coverage positions (Fig 1A and 1B). The same pattern was also observed in samples where mean coverage in the DNA Prep dataset was higher (Fig 1C and 1D). In cases where mean coverage was much higher in the Nextera XT prepared isolate, steep dips in coverage were observed which the DNA Prep dataset covered more evenly (Fig 1E and 1F). Coverage data for all isolates included in the study (n = 16) is provided in S1 and S2 Figs. Further analysis of the low coverage regions found that there was no relationship between low coverage regions and GC content when using either library preparation kit (Fig 2). Additionally, DNA Prep samples had fewer positions of zero coverage however this was not statistically significant (S3 Fig).

**Fig 3:**
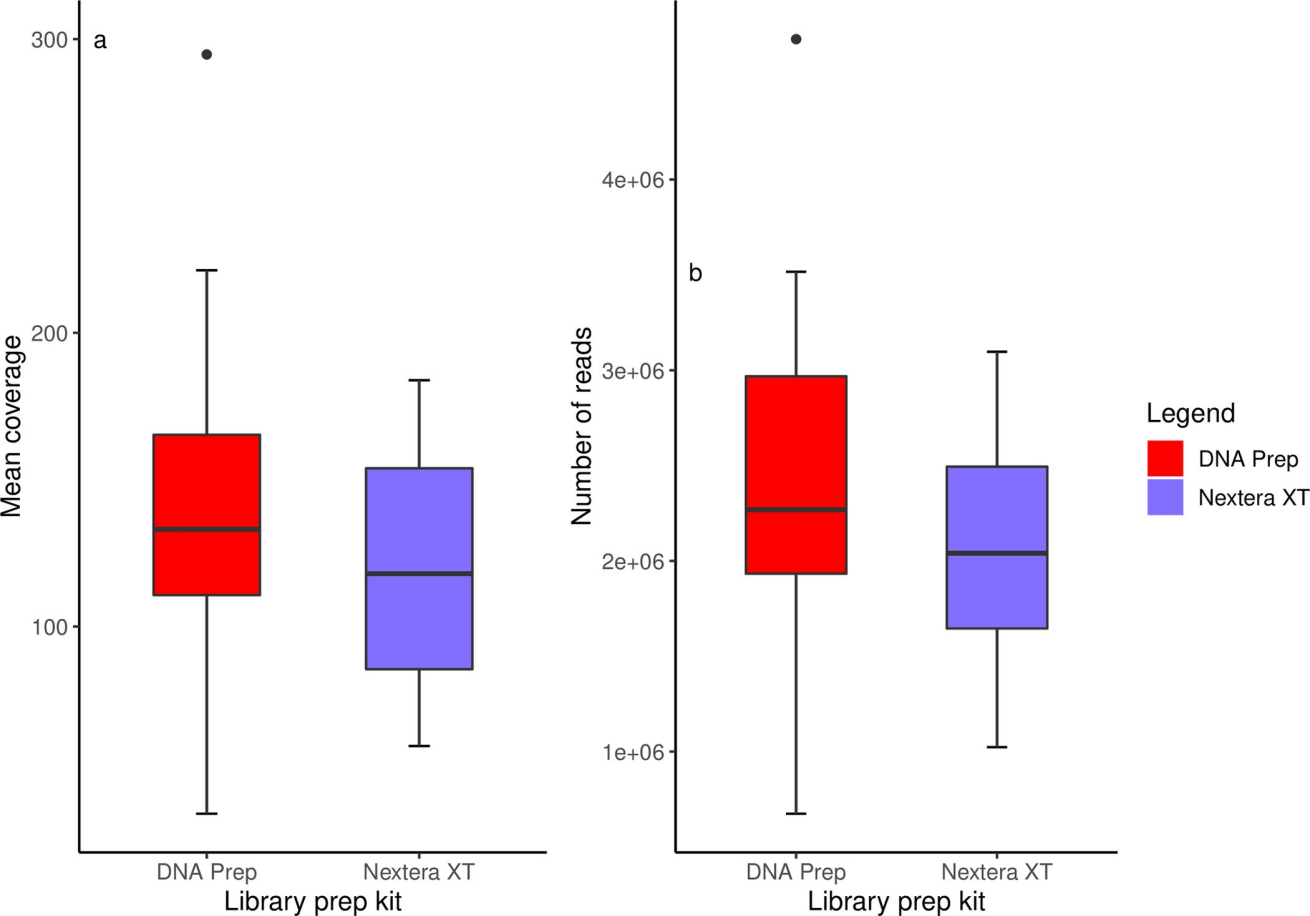
Comparison of mean sequencing coverage and number of reads per sample between the DNA Prep and Nextera XT kits. (A) Difference in mean sequencing coverage between the Nextera XT and DNA Prep kits, (B) Variation in the number of reads per sample between the Nextera XT and DNA Prep kits.

To explore whether the coverage bias findings may have been influenced by overall coverage differences or number of reads between each test group, a paired *t*-test was used to determine whether mean coverage or number of reads was impacted by which library preparation kit was used (Fig 3A and 3B). The average phred score across all samples was 34, therefore no reads from any sample were removed during quality control due to high quality of the sequencing run overall. A higher mean coverage was observed in DNA Prep samples (mean = 140.39, 95% CI [109.25, 171.53]) compared to their Nextera XT counterparts (mean = 117.49, 95% CI [98.64, 136.34]), although this was not statistically significant (*t* = -1.211, *df* = 15, *p* = 0.2446, *d* = -0.3027). A higher number of reads per FASTQ file was also observed across DNA Prep samples (mean = 2412785, 95% CI [1937443, 2888127]) compared to Nextera XT FASTQ files (mean = 2073048, 95% CI [1765140, 2380956]), though again this was not statistically significant (*t* = 1.0867, *df* = 15, *p* = 0.2943, *d* = -0.2717). This finding indicated that the coverage bias observed was not a result of uneven pooling or higher DNA input.

### 3.2 Average fragment size

**Fig 4:**
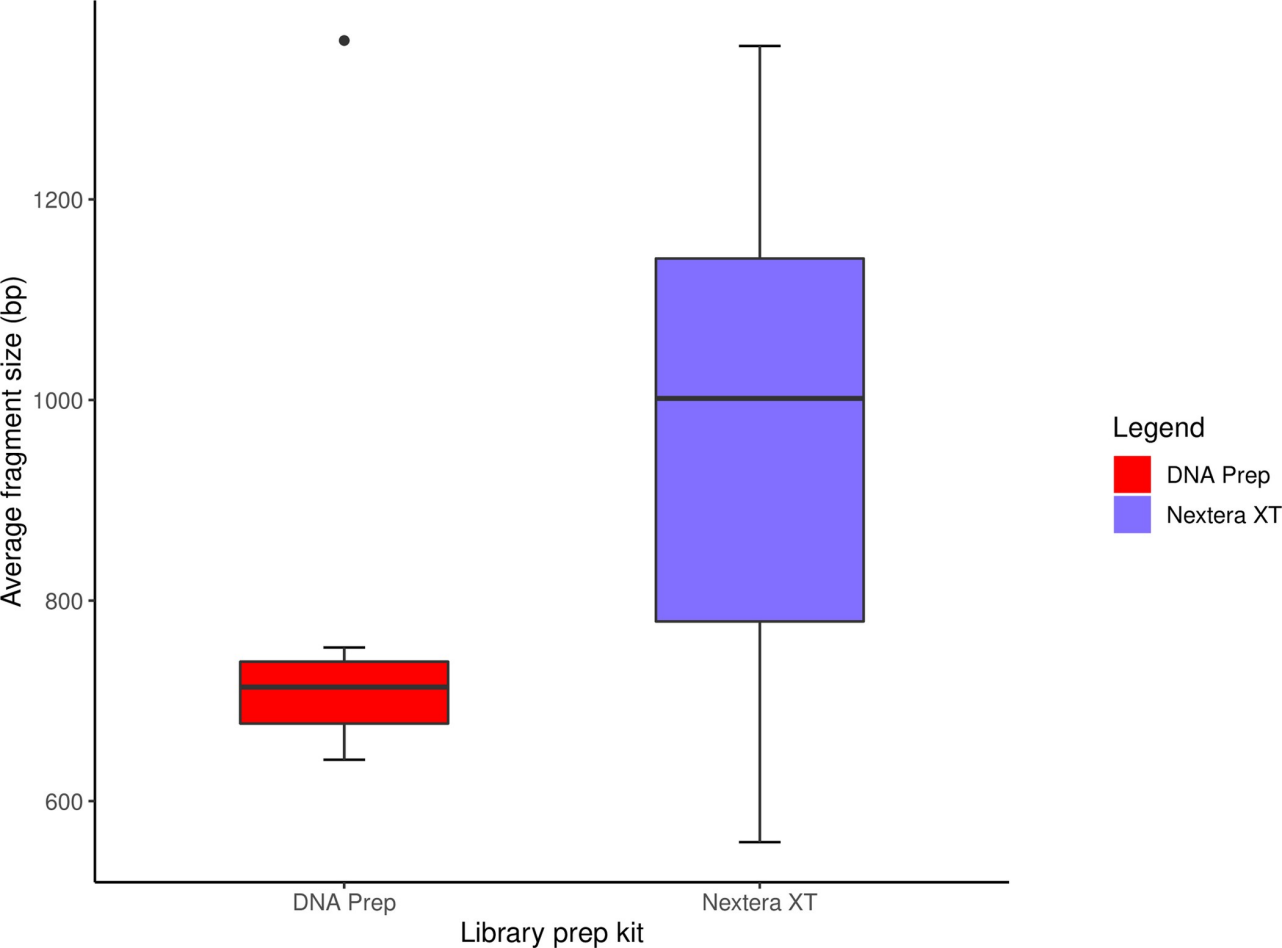
Comparison of average fragment size between the Nextera XT and DNA Prep kits. The DNA Prep kit yielded fragments with a smaller and more tightly distributed size range compared to the Nextera XT kit.

DNA Prep prepared isolates had a lower and more consistently distributed fragment length (mean = 746, 95% CI [663, 828]) compared to Nextera XT prepared isolates (mean = 965, 95% CI [854, 1077]) (Fig 4). The results of the paired *t*-test indicated that the difference in average fragment length between kits was statistically significant (*t* = -2.8354, *df* = 15, *p* = 0.01253, *d* = -0.7088).

### 3.3 Tagmentation bias

**Fig 5:**
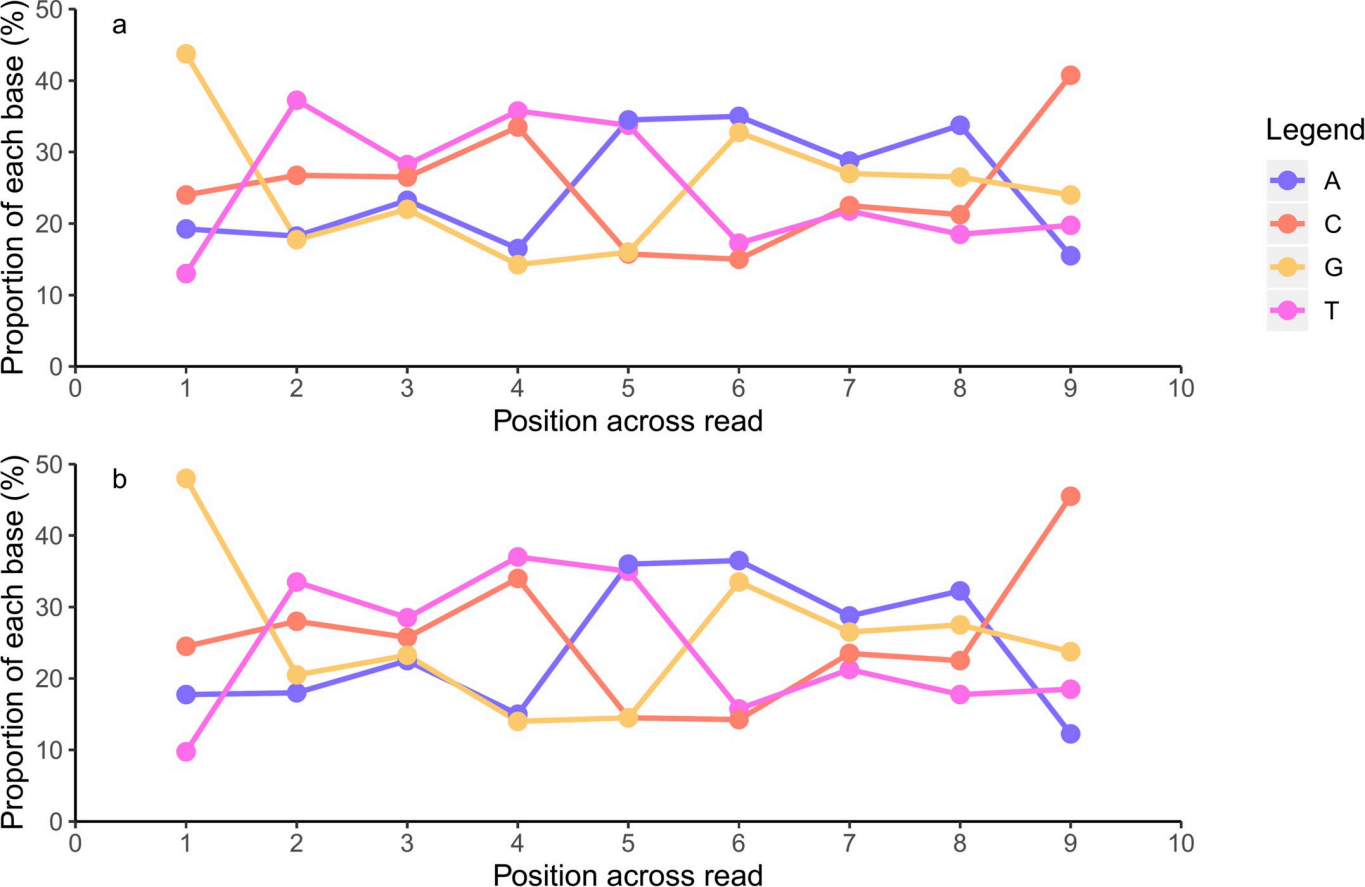
Tagmentation bias observed in both the Nextera XT and DNA Prep library preparation kits. (A, B) The average proportion of each of the four bases at the first nine positions of each read is displayed here (A: Nextera XT, B: DNA Prep), indicating that a highly similar sequence motif is preferentially tagmented by the transposome in both library preparation kits (5’-GTNYWRNAC).

To determine whether a tagmentation bias was present in either library preparation kit, the FastQC per base sequence content metric was used to evaluate the average proportion of each of the four bases at each position across all the reads in a FASTQ file (Fig 5) [24]. FASTQ files were merged based on which library preparation kit was used prior to analysis. As demonstrated in Fig 5, the results from this module indicate the transposase in both library preparation kits preferentially inserted into DNA with a 5’-GTNYWRNAC-like sequence motif.

### 3.4 *De novo* assembly quality

**Fig 6:**
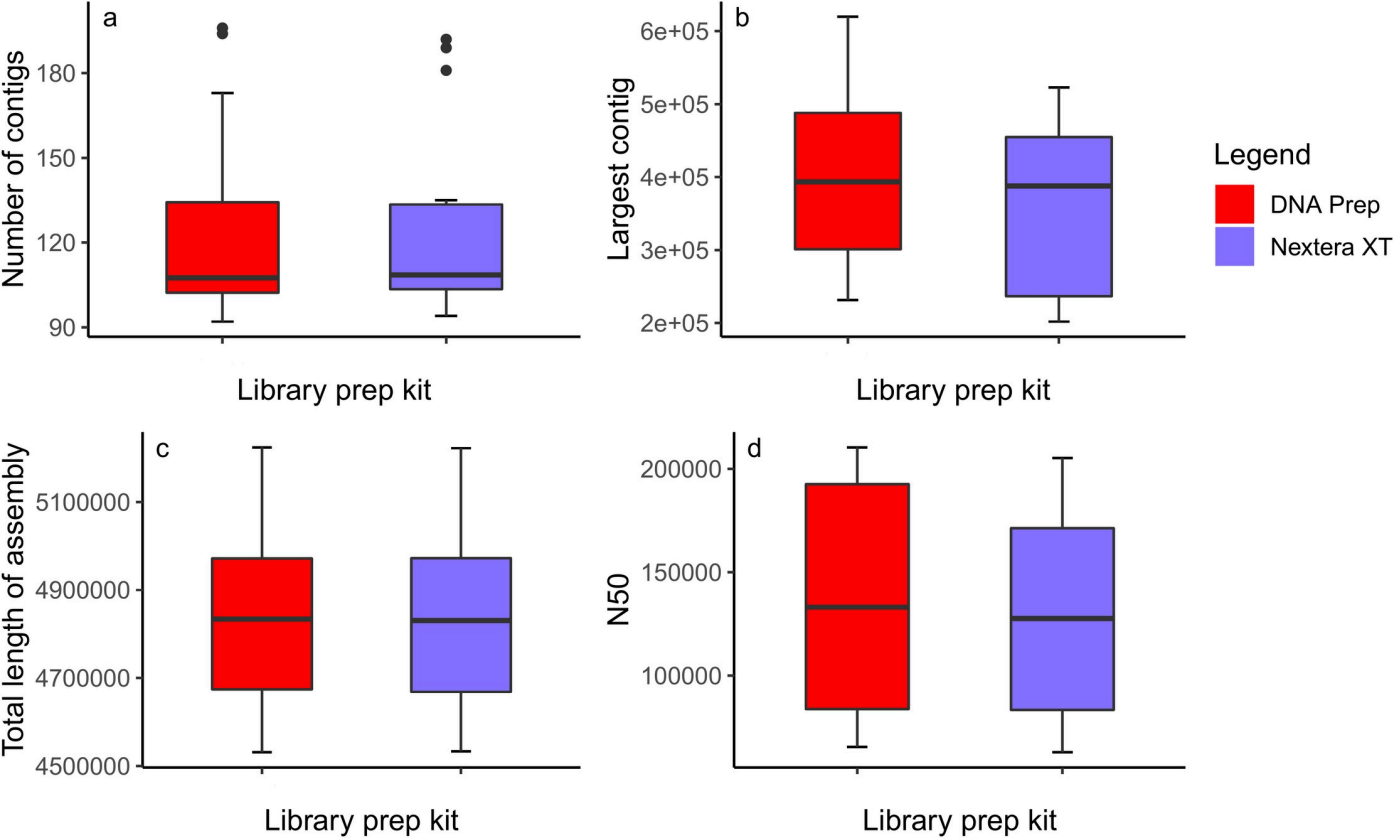
Comparison of *de novo* assembly quality between the two library preparation kits. (A-D) The difference in number of contigs per assembly (A), size of the largest contig in the assembly (B), total assembly length (C) and N50 based on which kit was used for library preparation.

Four measures were used to determine whether there were any notable differences in *de novo* assembly quality between the Nextera XT and the DNA Prep samples, which included largest contig in the assembly, total number of contigs, total assembly length and N50 (Fig 6). Overall there was no significant difference in assembly quality metrics between the library preparation kits. The largest contig in the assembly was larger in DNA Prep samples (mean = 400637, 95% CI[338334.3, 462939.7]) than in Nextera XT samples (mean = 363591, 95% CI[309802.5, 417379.5]) however this was not statistically significant (*t* = -1.487, *df* = 15, *p* = 0.1577, *d* = -0.3717). The number of contigs in DNA Prep assemblies (mean = 123.0, 95% CI[106.11, 139.89]) was very similar to Nextera XT assemblies (mean = 124.7, 95% CI[108.34, 141.06]), as was the total assembly length of DNA Prep assemblies (mean = 4851046, 95% CI[4742937, 4959155]) compared to their Nextera XT counterparts (mean = 4849838, 95% CI[4741605, 4958071]). The N50 of DNA Prep assemblies (mean = 136931, 95% CI[110266.8, 163595.2]) was also very similar to Nextera XT assemblies (mean = 130586, 95% CI[103921.8, 157250.2]).

## 4. Discussion

This study aimed to establish whether the DNA Prep kit reduced sequencing bias associated with the library preparation of samples undergoing whole-genome sequencing, when compared to the Nextera XT kit. While the DNA Prep kit did reduce coverage bias, a key finding of this research was that there was no relationship between low coverage and GC content among any of the samples in both Nextera XT and DNA Prep datasets. There have been several conflicting reports on the role of the Nextera XT kit in GC content bias in sequencing data. When the Nextera library preparation kit was first released, GC content bias was only reported in samples with low GC content (≤ 45%), and this was mitigated by a PCR-free protocol [8]. This was supported by observations of no GC content bias in GC-neutral samples such as in *Escherichia coli* genomes, and samples with low GC content having profound coverage bias [9, 10]. Others have found no substantial differences in coverage bias when using the Nextera XT kit compared to other market leaders with both PCR and PCR-free protocols [11–13], while significant differences have been noted in coverage bias, with the Nextera XT kit performing unfavourably when compared to the PCR-free systems [14–16]. Recent comparisons of the Nextera XT and DNA Prep kits have found significant inadequacies of the former due to GC content-related bias [10, 17]. It has been suggested that the Nextera XT kit contributed to errors in serotyping *Salmonella* (namely detection of the O antigen) due to poor coverage of gene clusters with low GC content (≈ 30%) despite the average genomic GC content of *Salmonella* spp. being approximately 52% [18–20]. Approximately 0.058% of the *E*. *coli* genome contains GC-rich sequences (≥75%) with the overall base composition of the genome being GC neutral [32], potentially explaining the lack of GC content bias observed in the present study.

Using Illumina sequencing chemistry, amplification bias can result from PCR amplification during library preparation and cluster generation on the flow cell [7]. Difficulties sequencing GC-rich [21, 33, 34], and GC-poor sequences [35–37], and more specifically AT-dinucleotide repeats, poly-G and poly-C homopolymers have been widely reported [32]. Some have found that amplification of AT-rich sequences can be enhanced by choice of polymerase used during PCR [38, 39–42], while others have found this not to be the case [16]. Strong advocates have emerged for either PCR-free library preparation or low-cycle number PCR amplification to reduce bias, noting that coverage bias still occurred in PCR-free libraries [11, 32, 38, 43, 44–49].

The Nextera XT system utilised a hyperactive Tn*5* transposase that enabled fragmentation and adapter ligation in a single step [8], with the DNA prep kit introducing a novel bead-linked transposase based on this enzyme [22]. This study assessed the potential for biased insertion of both transposases using the FastQC per base sequence content metric, which plotted the average proportion of each of the four bases at each position across all the reads in a FASTQ file [24]. The results from this study suggested that the transposase used in both library preparation kits preferentially targeted the same sequence motif 5’-GTNYWRNAC (Fig 5). While wild type Tn*5* transposases do have low target specificity [50], Goryshin *et al*. (1998) [51] reported the consensus target sequence of the wild type Tn*5* transposase as 5’-A-GNTYWRANC-T. Initial comparisons of transposase-mediated enzymatic fragmentation with mechanical and endonuclease-mediated fragmentation found that the Nextera transposase had low levels of insertion bias, targeting a motif that weakly resembled the wild type [8]. Subsequent reports found that the Nextera XT transposase had a preference for inserting in AT-rich regions, however no negative consequences were detected downstream as a result [16, 21]. A contrary report has stated that the insertion bias of the Nextera transposase is significant, and observed difficulties sequencing genomes that did not contain many copies of the 5’-A-GNTYWRANC-T consensus sequence with the Nextera XT kit due to inefficient tagmentation [14]. While the details of the changes to the transposase used in the DNA Prep kit outside of bead-linking are unknown [22], the results of this study suggest that the novel DNA Prep kit enzyme does not resolve the issues associated with insertion bias that were frequently reported with the Nextera XT kit.

This study found that the DNA Prep kit produced DNA libraries with a more consistent fragment size distribution than the Nextera XT kit. One of the most important outcomes of library preparation is ensuring that fragment size is optimal for efficient cluster generation [7]. During cluster generation, fragments that are too short cluster very efficiently and reduce the amount of useful data generated from the sequencing by synthesis reaction. Fragments that are too long are also suboptimal because they do not amplify efficiently and produce sparse clusters, which also reduces the amount of useful data yielded from the reaction [52]. One of the main differences between the Nextera XT and DNA Prep kits was the bead-linked transposase, reported to produce more consistent insert lengths during tagmentation. Following library amplification with DNA Prep, a more comprehensive clean-up using double-sided solid phase reversible immobilisation (SPRI) beads removes low and high molecular weight fragments to tighten the size distribution of the library [22]. Based on the results of the present study, the combination of the bead-linked transposases and double-sided SPRI clean-up was indeed effective in tightening the size distribution of each DNA library when using the DNA Prep library preparation kit. This may have had a role in reducing coverage bias by increasing cluster generation efficiency and ensuring more even representation of the library.

There was little improvement in the *de novo* assembly produced with the DNA Prep prepared samples based on number of contigs, size of the largest contig, total assembly length or N50, despite more even sequencing coverage. This finding was in line with the outcomes of a study by Huptas *et al*. (2016) [33] which found that factors such as which assembler was used for *de novo* assembly and read and insert lengths had a more substantial influence on assembly quality than sequencing depth. Despite this, it has been claimed that Nextera XT libraries produce particularly poor *de novo* assemblies [11]. In this study, the size of the largest contig appeared to be slightly higher on average in DNA Prep samples, which may be beneficial in bacterial typing where typing regions contain repeats.

Despite improved coverage uniformity when using the DNA Prep kit, a strong advantage of using the Nextera XT kit is the superior cost-effectiveness. Previous studies have found that using 25–50% of the recommended amounts of library preparation reagents, and in some cases as low as one-eighth of the recommended amount, can maximise the number of libraries produced with a single Nextera XT kit with no negative downstream effects on depth of coverage [12, 16, 41, 53, 54]. Similar benefits have yet to be reported using the DNA Prep kit.

## 5. Conclusions

This study has used a methodological approach to investigate the DNA Prep library preparation kit for sequencing of *E*. *coli* isolates, and found it to be advantageous where coverage bias is a major concern. It must be noted that despite the Nextera XT kit producing libraries with more coverage bias, based on our data, the differences in *de novo* assembly were not statistically significant. The improvements to sequencing coverage observed with the DNA Prep library preparation kit appeared unrelated to GC content.

Assessment of a more diverse set of input genomes with both extremely high and extremely low GC content will allow more sound conclusions to be made regarding the role of GC content bias. While the issues associated with the Nextera XT library preparation kit can be partially circumvented by increasing the concentration of DNA applied to the flow cell, the DNA Prep library preparation kit may be preferable where expected mean coverage is low, potentially allowing more samples to be pooled and sequenced on the same run without major impact on downstream analyses. Based on the results of this study it is concluded that DNA Prep offers minor benefits over Nextera XT, however whether these benefits are sufficient to warrant laboratories switching established workflows is questionable, particularly when studying GC-neutral organisms.

## Supporting information

S1 Fig Differences in uniformity of coverage between the Nextera XT and DNA Prep kits (n = 16). On the left, coverage count data is displayed as frequency and on the right, coverage count data is displayed as bar plots. Nextera XT data is shown in blue and DNA Prep data is shown in red. Overlapping data points appear purple.
(PDF)

S2 Fig The percentage of the *Escherichia coli* K12 reference genome covered by the Nextera XT and DNA Prep datasets (n = 16). Bar plots indicate the percentage of the reference genome covered by each library preparation kit at depths ranging from 0–600. Nextera XT data is shown in blue and DNA Prep data is shown in red. Overlapping data points appear purple.
(PDF)

S3 Fig Difference in number of positions of zero coverage across Nextera XT and DNA Prep datasets (n = 16). Boxplots illustrate the distribution of the number of zero coverage positions across the Nextera XT (blue) and DNA Prep (red) samples. The DNA Prep samples had a lower number of zero coverage positions when aligned to the *Escherichia coli* K12 reference genome, however this was not statistically significant (data not shown).
(PDF)
