# Supplementary material for: Evaluating coverage bias in next-generation sequencing of Escherichia coli
Source: PLoS One. 2021 Jun 24;16(6):e0253440. doi: 10.1371/journal.pone.0253440 (PMC8224930; doi:10.1371/journal.pone.0253440)

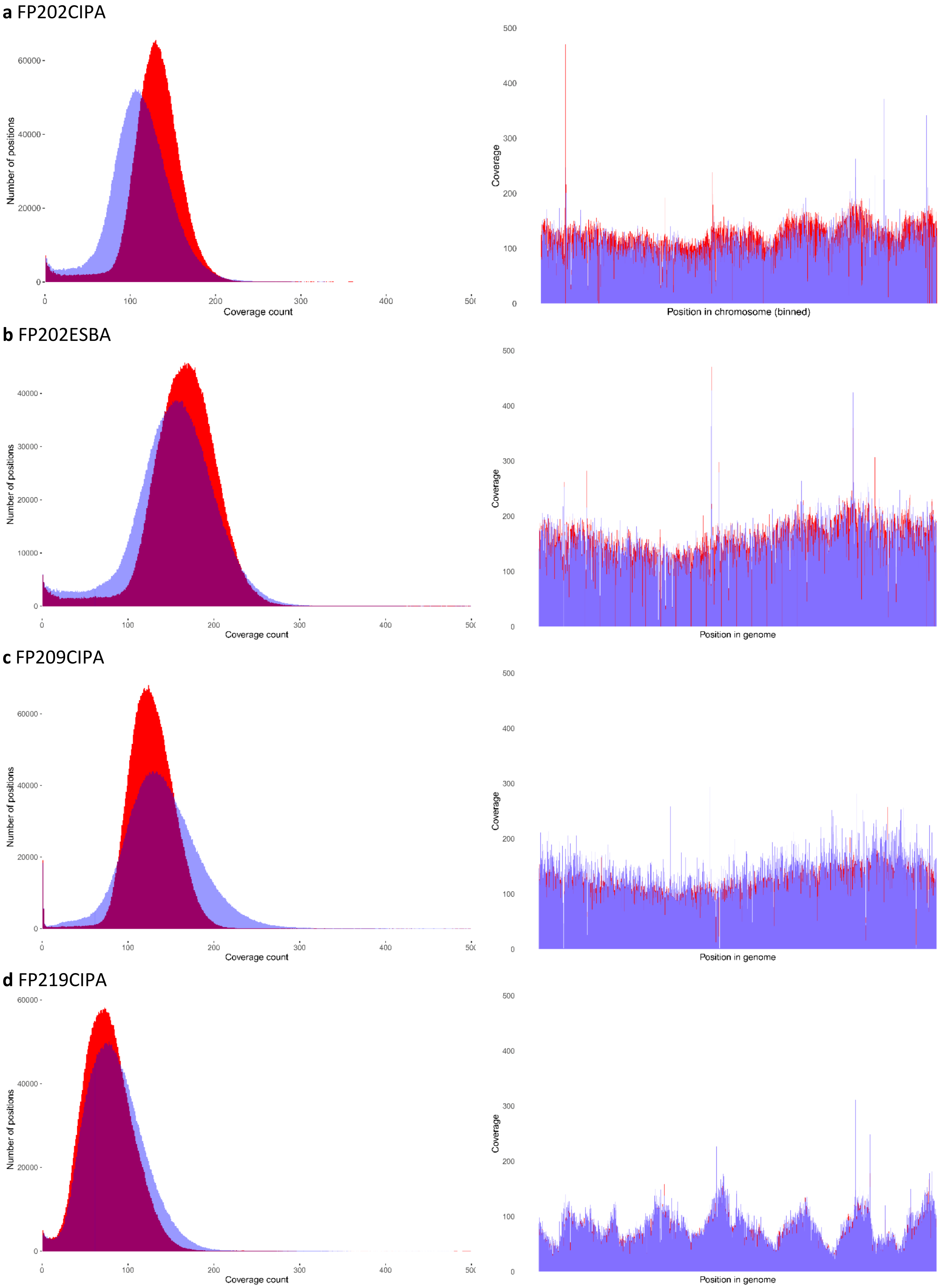

a FP202CIPA
Number of positions
Coverage count
Coverage
Position in chromosome (binned)
b FP202ESBA
Number of positions
Coverage count
Coverage
Position in genome
c FP209CIPA
Number of positions
Coverage count
Coverage
Position in genome
d FP219CIPA
Number of positions
Coverage count
Coverage
Position in genome

e FP219ESBA

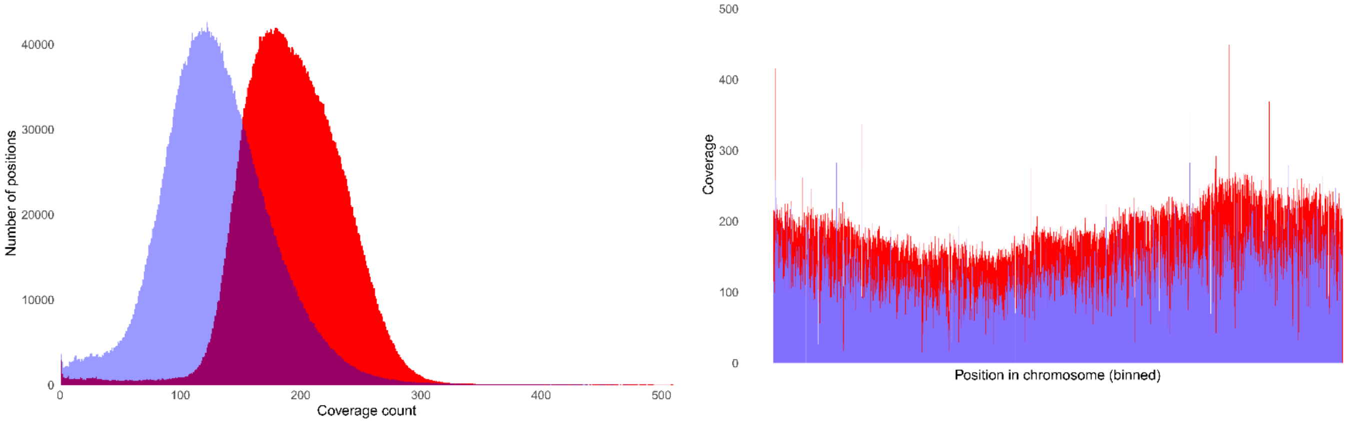

f FP222CIPA

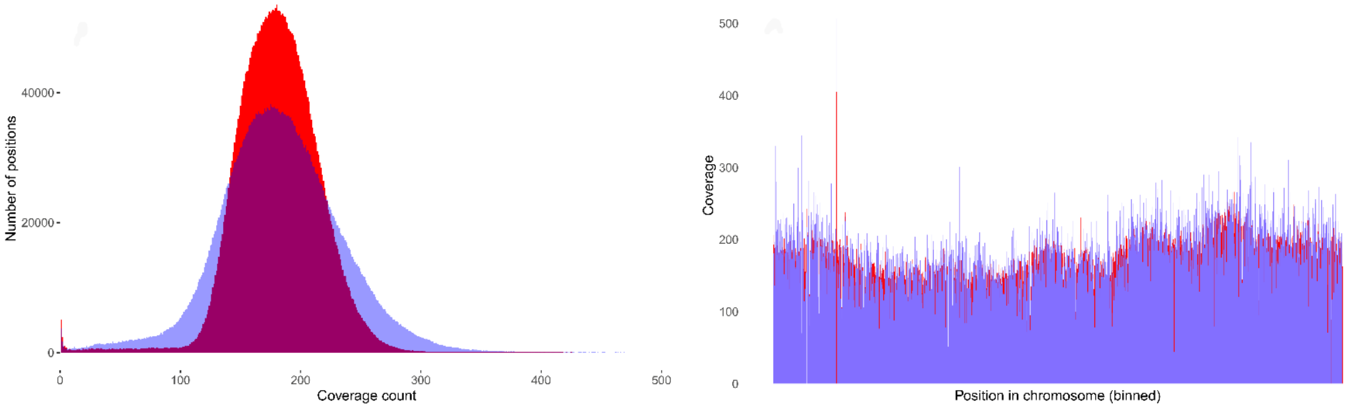

g FP222ESBA

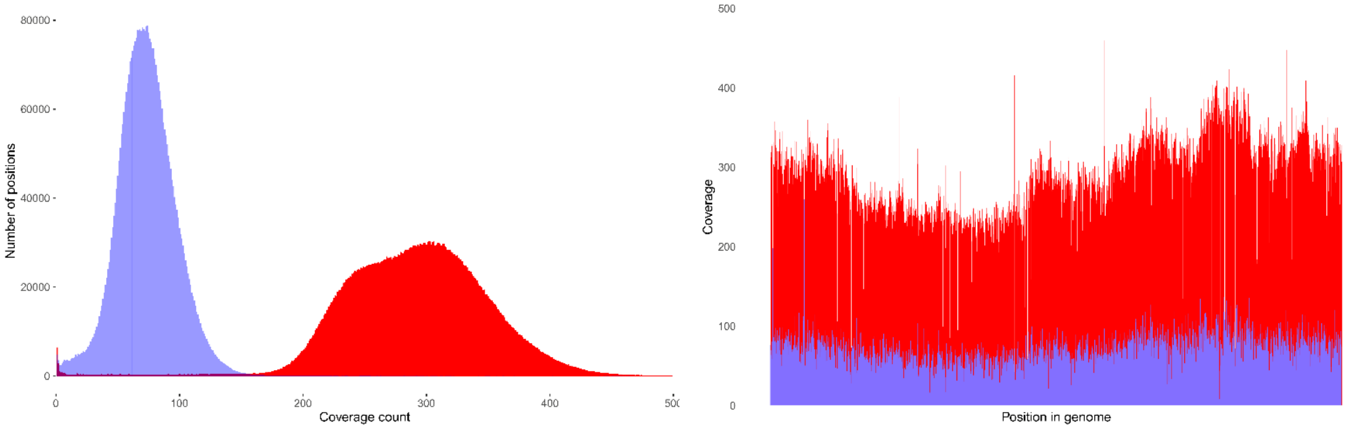

h FP225CIPA

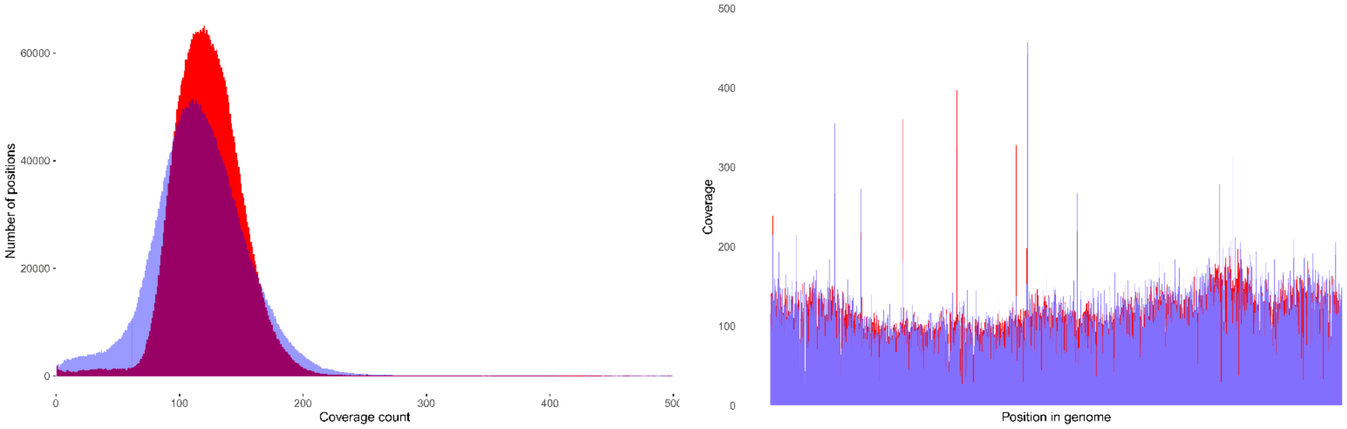

i LP143CIPA

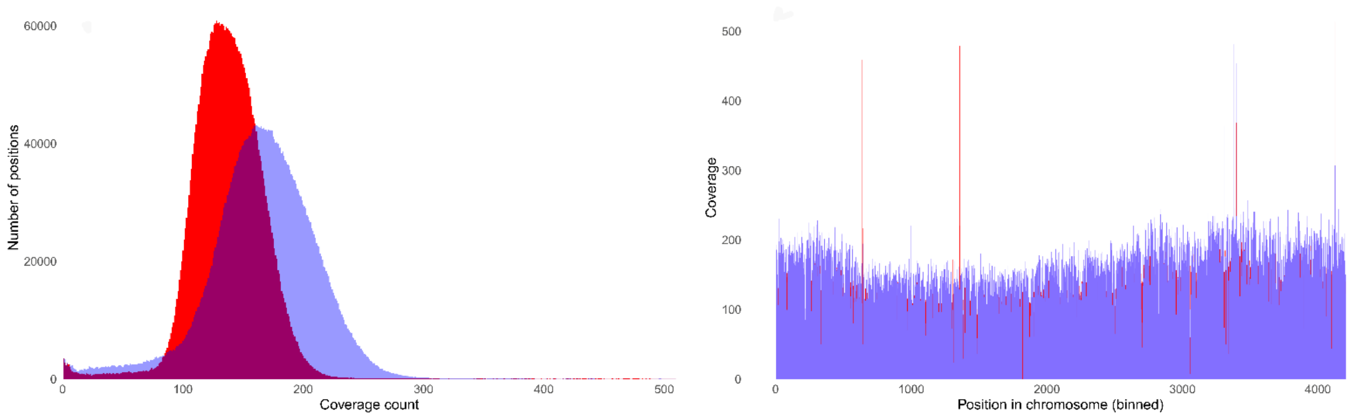

j LP143ESBA

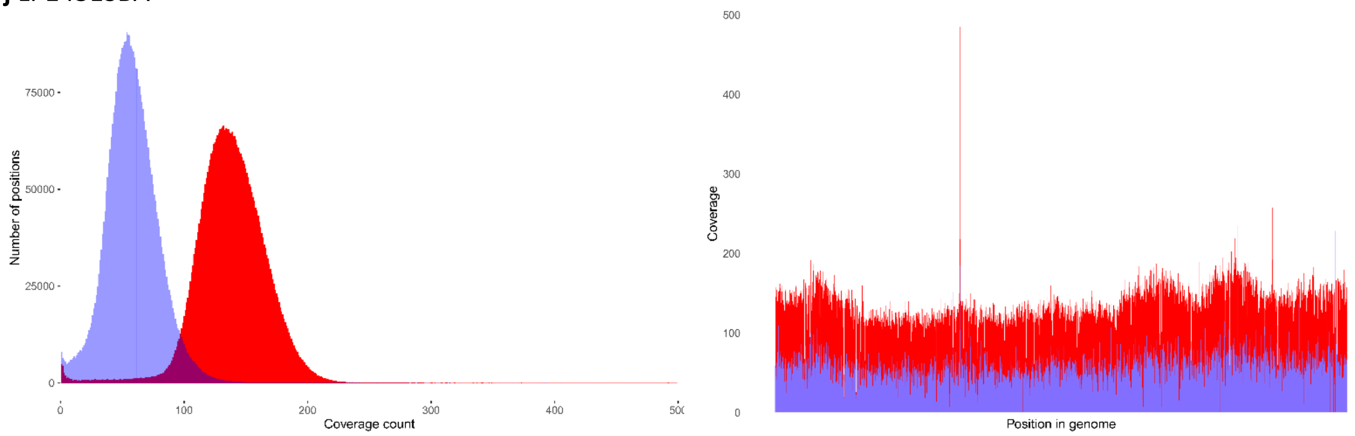

k LP233CIPB

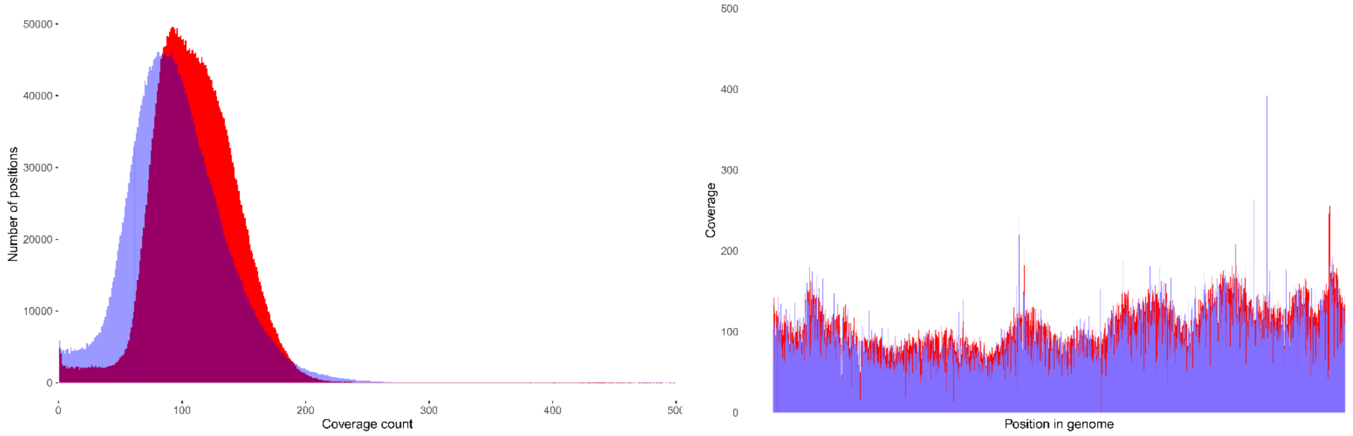

l LP233ESBA

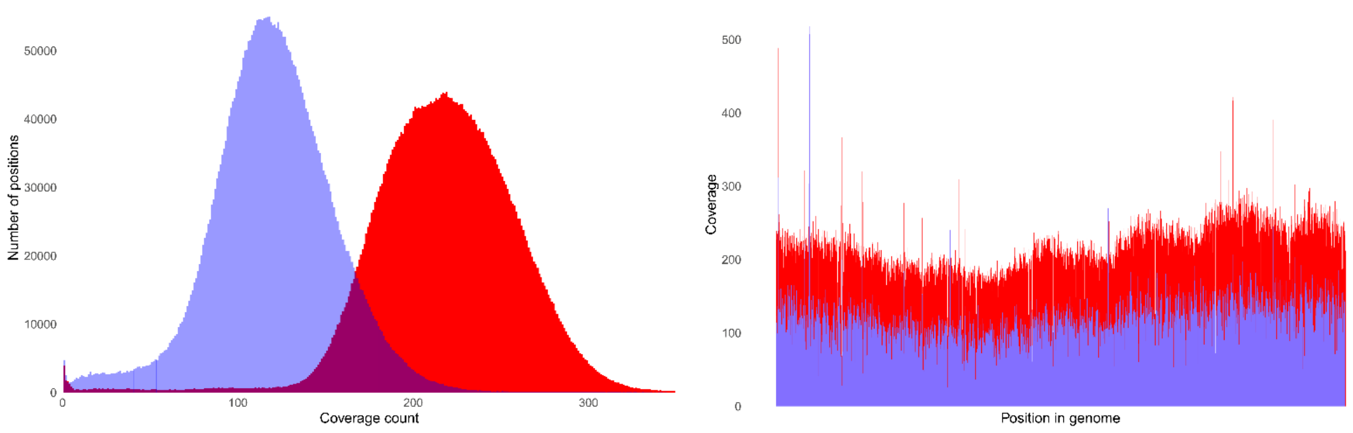

m LP239CIPA

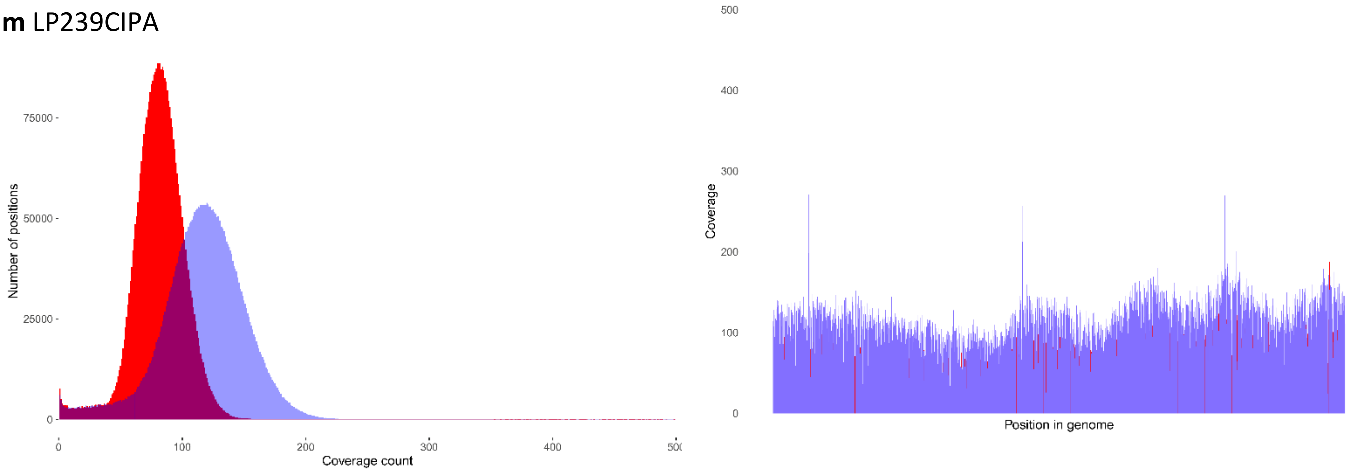

n LP249ESBA

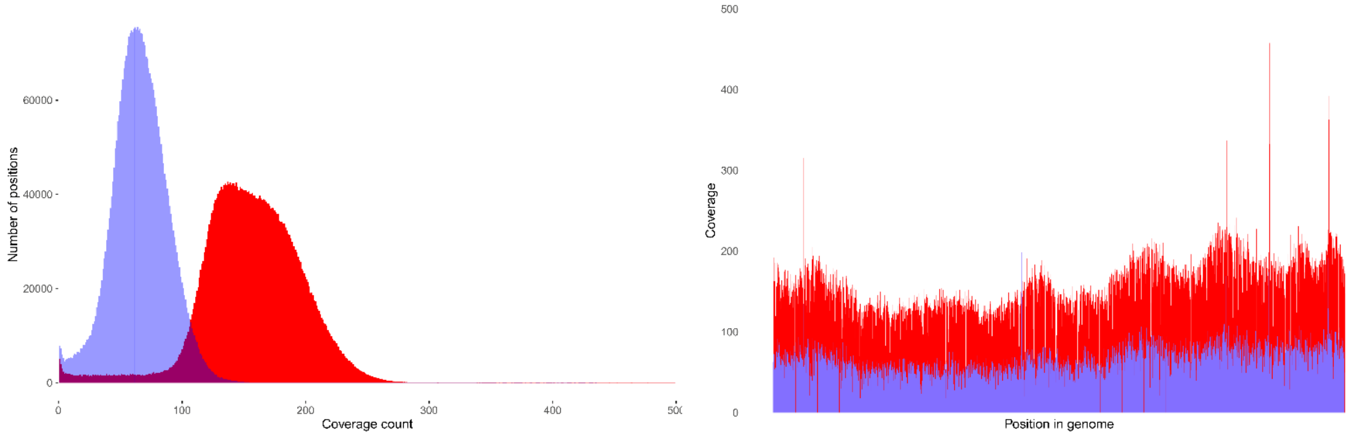

o LP250ESBA

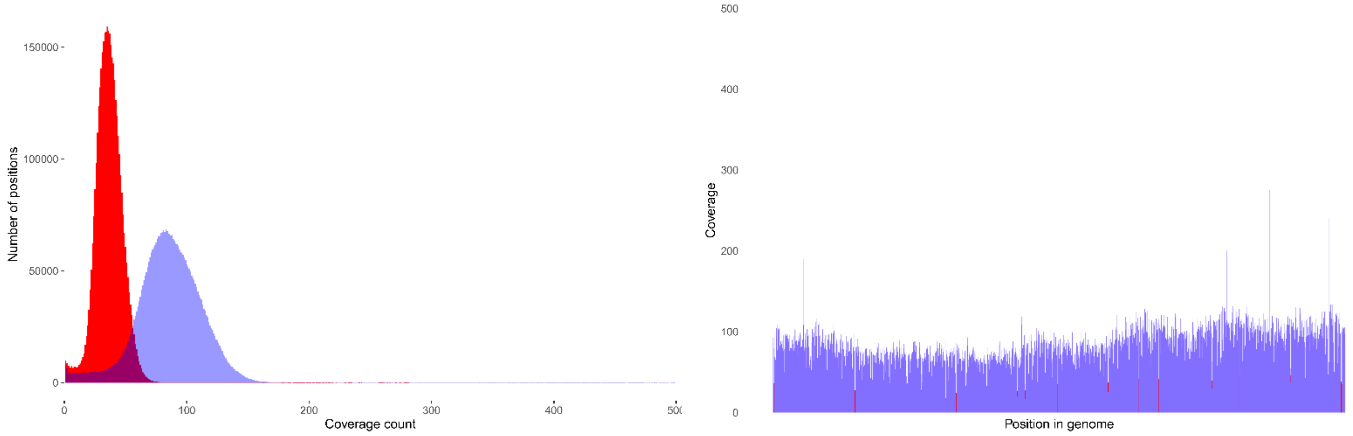

p LP251CIPA

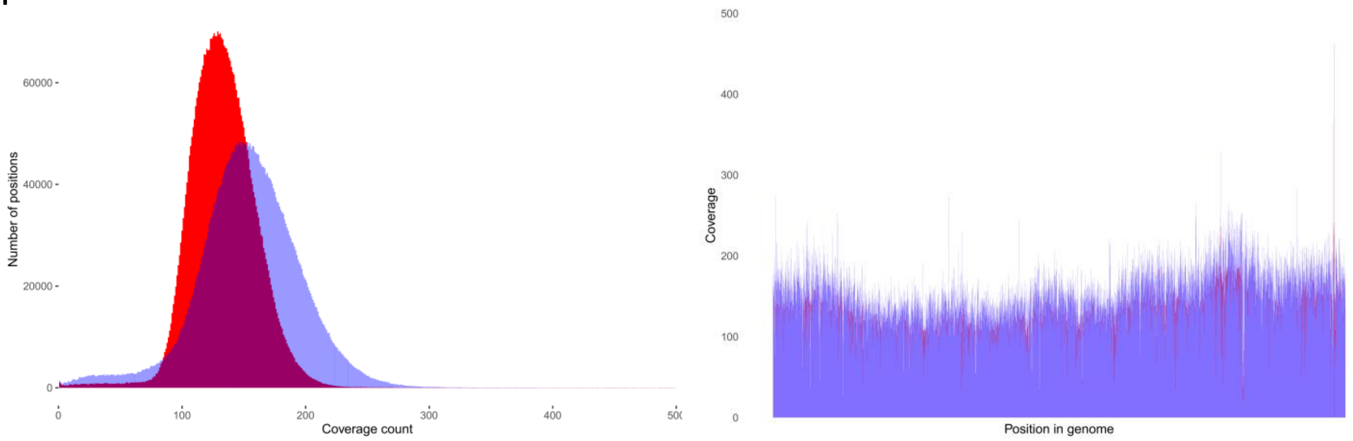

Supplement: S1 Fig — On the left, coverage count data is displayed as frequency and on the right, coverage count data is displayed as bar plots. Nextera XT data is shown in blue and DNA Prep data is shown in red. Overlapping data points appear purple. (PDF) [file pone.0253440.s001.pdf]
