# Supplementary material for: Evaluating coverage bias in next-generation sequencing of Escherichia coli
Source: PLoS One. 2021 Jun 24;16(6):e0253440. doi: 10.1371/journal.pone.0253440 (PMC8224930; doi:10.1371/journal.pone.0253440)

a FP202CIPA

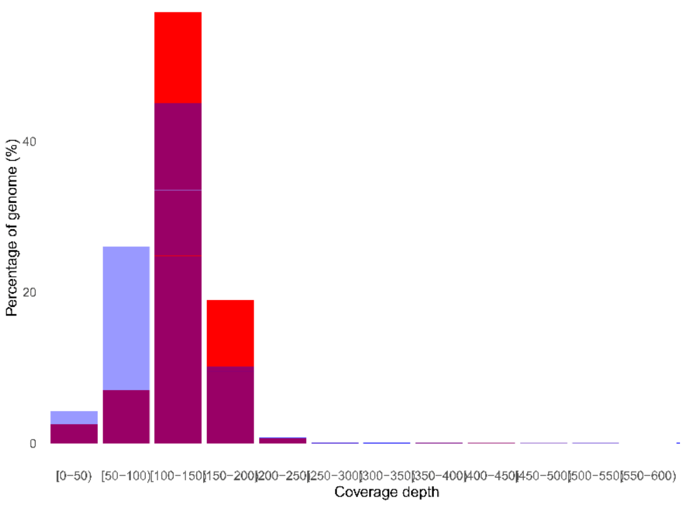

d FP219CIPA

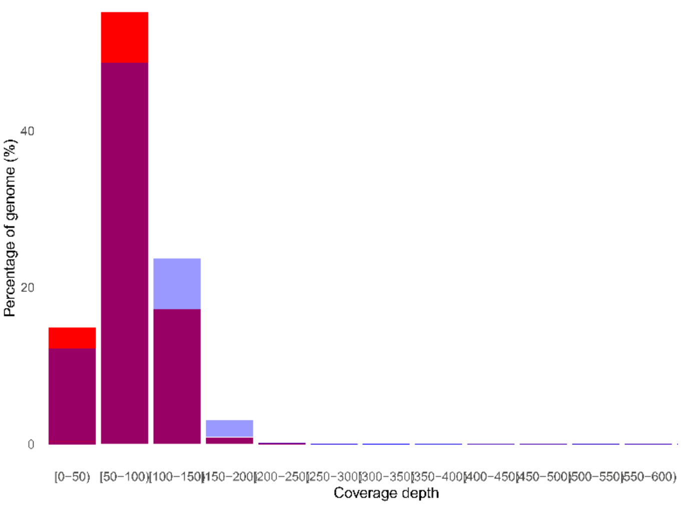

b FP202ESBA

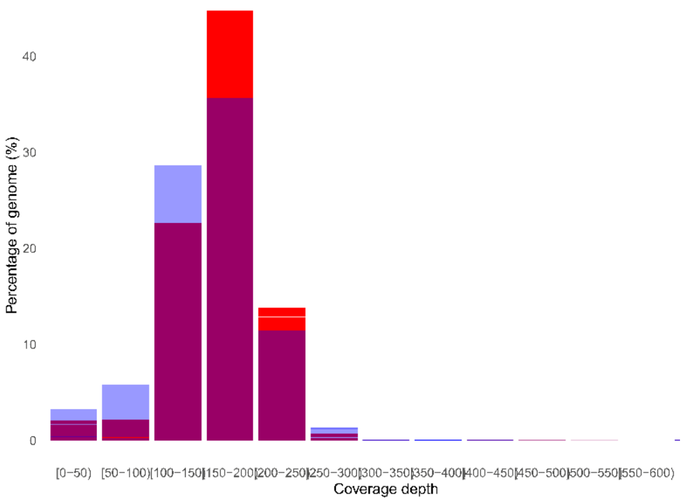

e FP219ESBA

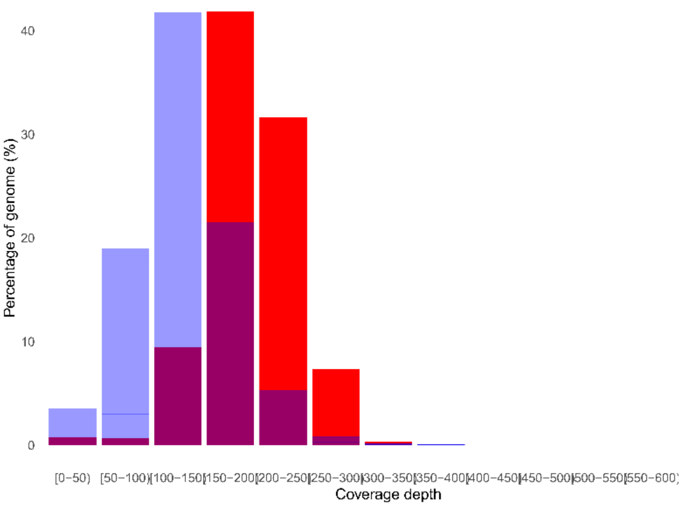

c FP209CIPA

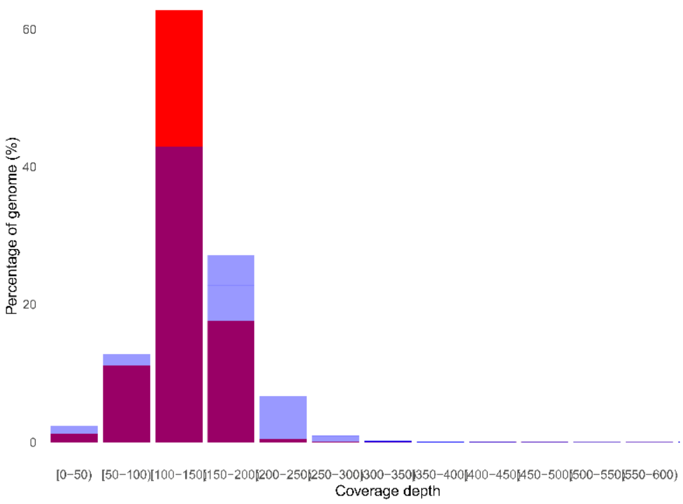

f FP222CIPA

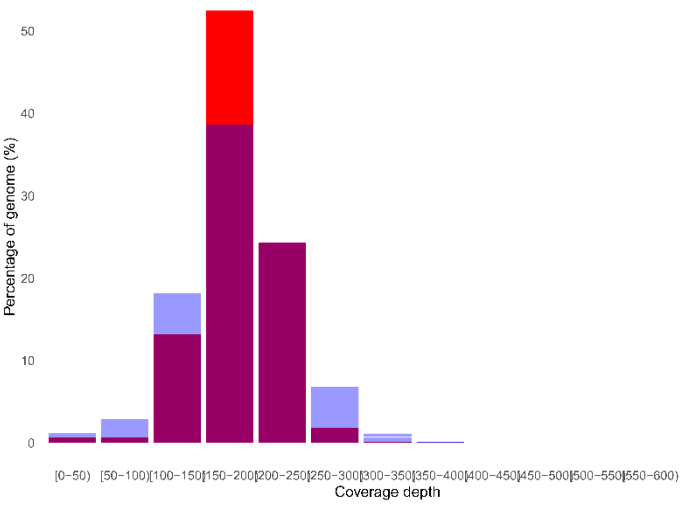

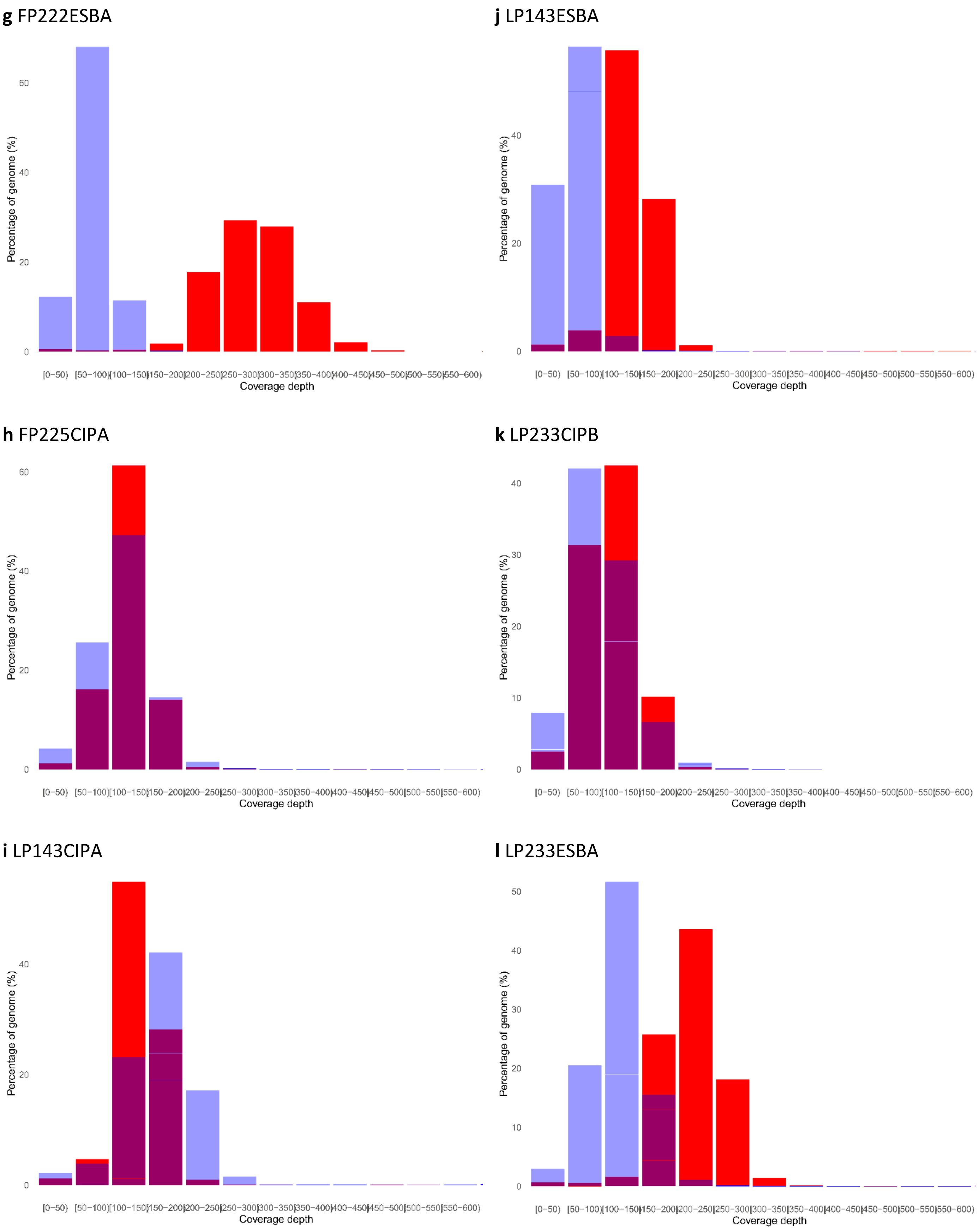
g FP222ESBA
j LP143ESBA
h FP225CIPA
k LP233CIPB
i LP143CIPA
l LP233ESBA
Percentage of genome (%)
Coverage depth
[0–50)
[50–100)
[100–150)
[150–200)
[200–250)
[250–300)
[300–350)
[350–400)
[400–450)
[450–500)
[500–550)
[550–600)
0
10
20
30
40
50
60

m LP239CIPA

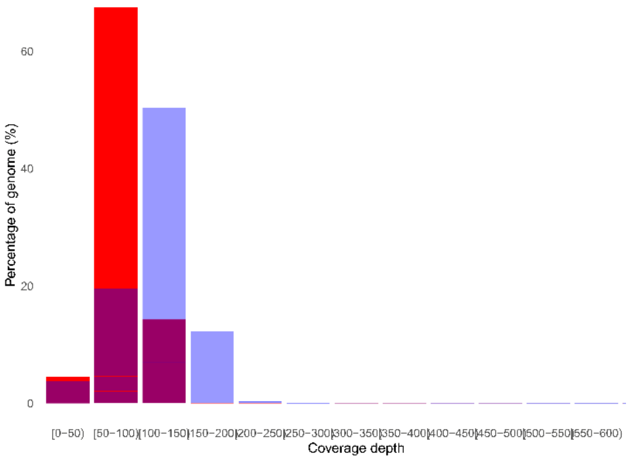

p LP251CIPA

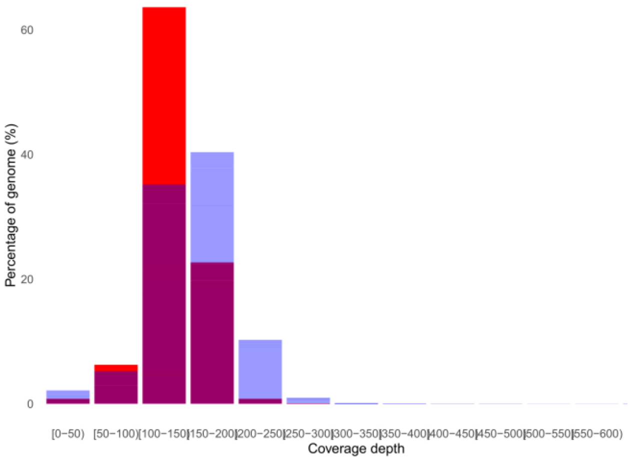

n LP249ESBA

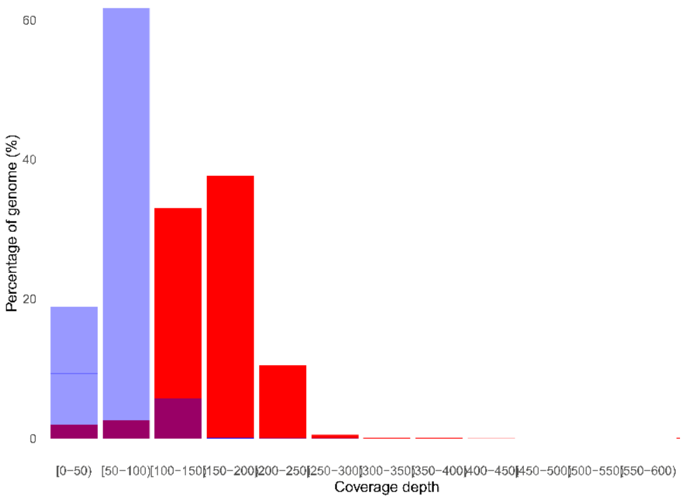

o LP250ESBA

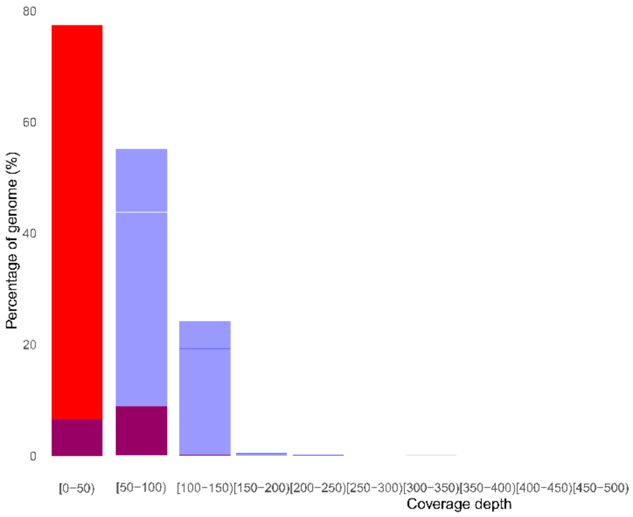

Supplement: S2 Fig — Bar plots indicate the percentage of the reference genome covered by each library preparation kit at depths ranging from 0–600. Nextera XT data is shown in blue and DNA Prep data is shown in red. Overlapping data points appear purple. (PDF) [file pone.0253440.s002.pdf]
