# Supplementary figures and images for: Evaluating coverage bias in next-generation sequencing of Escherichia coli
Source: PLoS One. 2021 Jun 24;16(6):e0253440. doi: 10.1371/journal.pone.0253440 (PMC8224930; doi:10.1371/journal.pone.0253440)

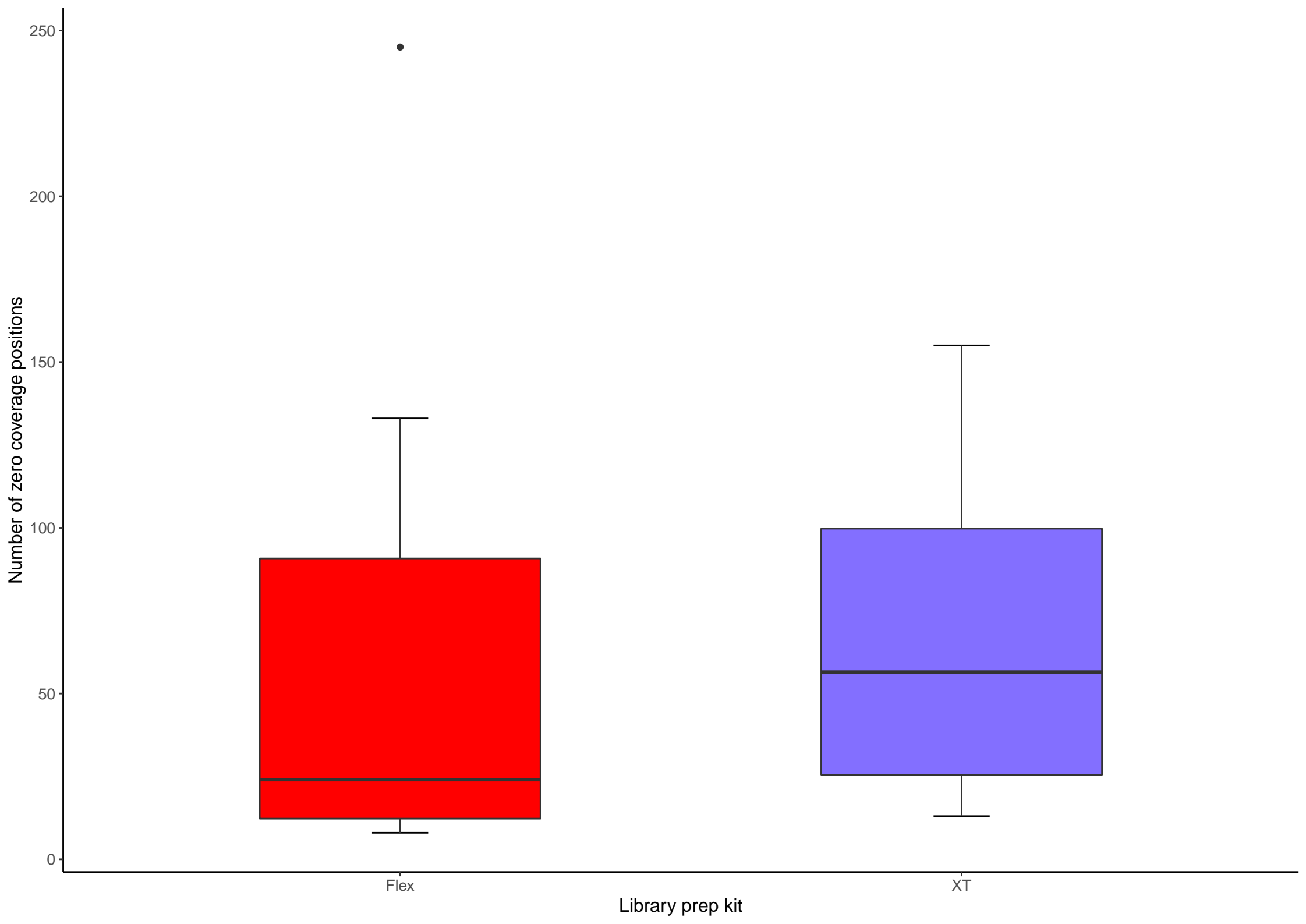

Supplement: S3 Fig — Boxplots illustrate the distribution of the number of zero coverage positions across the Nextera XT (blue) and DNA Prep (red) samples. The DNA Prep samples had a lower number of zero coverage positions when aligned to the Escherichia coli K12 reference genome, however this was not statistically significant (data not shown). (PDF) [file pone.0253440.s003.pdf]
